# Supplementary material for: Structure and Function of BcpE2, the Most Promiscuous GH3-Family Glucose Scavenging Beta-Glucosidase
Source: mBio. 2022 Aug 1;13(4):e00935-22. doi: 10.1128/mbio.00935-22 (PMC9426481; doi:10.1128/mbio.00935-22)
Supplement: FIG S1 [file mbio.00935-22-s0001.docx]

**Supplementary Figure S1. Structure-based sequence alignment of BcpE2 and its closest characterized GH3 enzymes.**

The two catalytic residues – Asp239 and Glu582 – are strictly conserved in all homologous GH3s. The active site of BcpE2 also includes Asp59, Arg127, and three additional aromatic AAs, namely Tyr207, Trp240, and Phe499 which are generally conserved. Asp59 and Arg127 show perfect conservation among all GH3 enzymes displayed in Figure S1, and Trp240 was systematically found next to the catalytic aspartate. Tyr207 was found in all sequences except in BglI of *Schwanniomyces etchellsii* which presented a leucine residue instead. The important Phe499 residue was shared by most GH3-family enzymes considered but sometimes aligned at an adjacent position as in BglI of *S. etchellsii* and KmBglI of *K. marxianus*. Only DesR from *S. venezuelae* and Bgl3B from *Cellulomonas fimi* did not display any phenylalanine residue in this subregion.


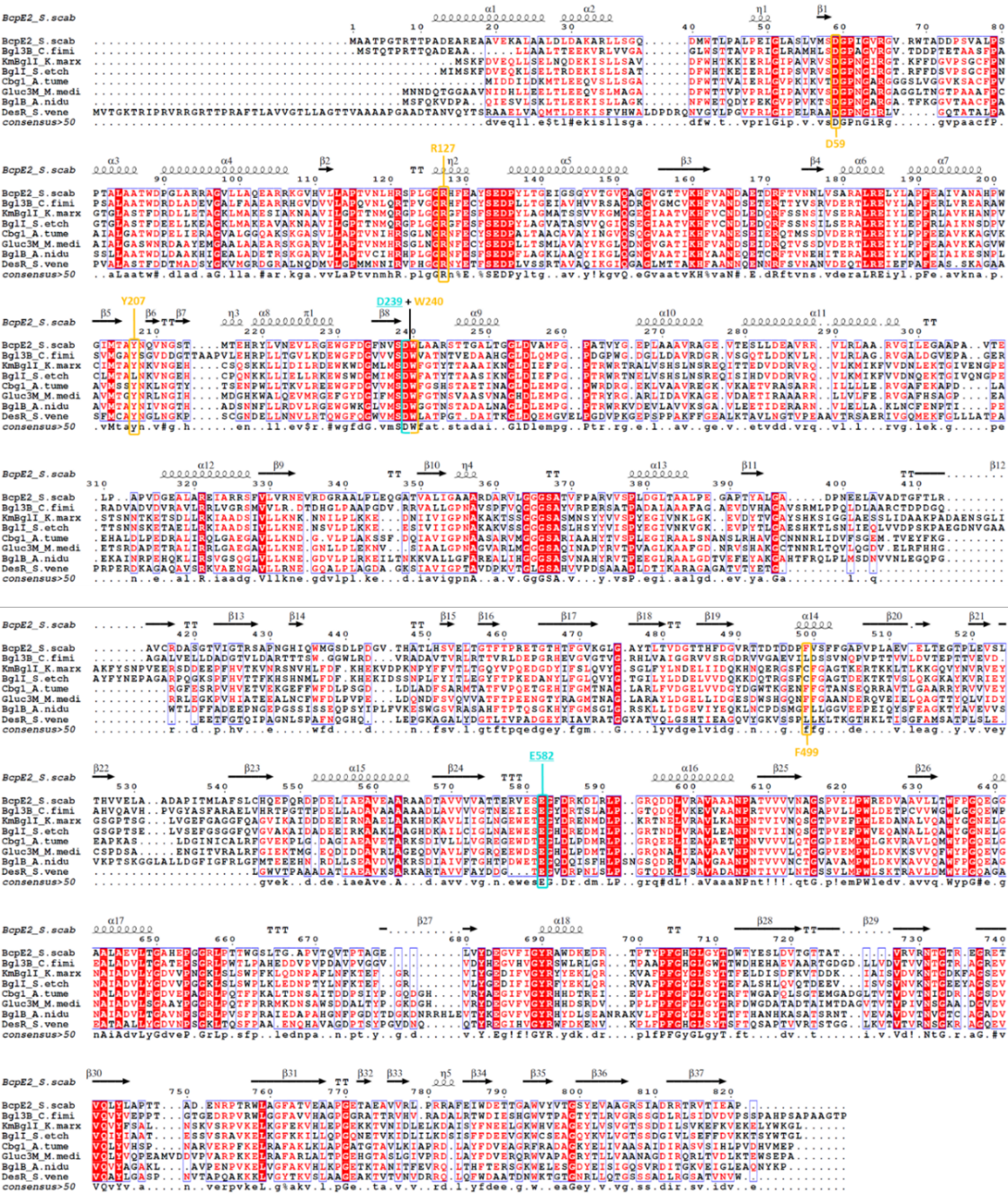


**Figure S1. Structure-based sequence alignment of BcpE2 and its closest characterized GH3 enzymes.** The residues involved in the active site are indicated in yellow and the catalytic residues, also in the active site, are highlighted in cyan. The red color on residues either indicate perfect conservation (red background) or biochemical similarity (red letter). Blue frames highlight conserved regions for which a consensus motif is found. The “!”, “#”, and “%” symbols in the consensus sequence shows the conservation of branched-chain, acidic or amide, and hydrophobic amino acids (AAs), respectively.
