# Supplementary material for: Structure and Function of BcpE2, the Most Promiscuous GH3-Family Glucose Scavenging Beta-Glucosidase
Source: mBio. 2022 Aug 1;13(4):e00935-22. doi: 10.1128/mbio.00935-22 (PMC9426481; doi:10.1128/mbio.00935-22)
Supplement: TABLE S1 [file mbio.00935-22-s0004.docx]

**Supplementary Table S1**

**Table S1 - Characteristics of GH3-family proteins homologous to BcpE2 (used for structure-based alignment and phylogenetic tree construction)**

| **Organism** | **GH3 name** | **Query cov. (%)** | **Identity (%)** | **PA14 domain** | **NCBI**  **protein ID** | **Ref.** |
| --- | --- | --- | --- | --- | --- | --- |
| *Streptomyces phaeolivaceus* | BcpE2-like | 99 | 86 | Yes | WP_152168420.1 | - |
| *Streptomyces diastatochromogenes* | BcpE2-like | 99 | 80 | Yes | WP_094217992.1 | - |
| *Streptomyces venezuelae* | BcpE2-like | 99 | 75 | Yes | WP_150183929.1 | - |
| *Streptomyces rimosus* | BcpE2-like | 96 | 66 | Yes | WP_033029078.1 | - |
| *Streptomyces coelicolor* | BcpE2-like? | 96 | 47 | Not found | WP_011031033.1 | - |
| *Cellulomonas fimi* | Bgl3B | 96 | 44 | Not found | AEE44608.1 | (Gao & Wakarchuk, 2014) |
| *Agrobacterium tumefaciens* | Cbg1 | 97 | 39 | Yes | AAA22082.1 | (Castle et al., 1992) |
| *Martelella mediterranea* | Gluc3M | 95 | 39 | Yes | ADC53302.1 | (Mao et al., 2010) |
| *Aspergillus nidulans* | BglB | 97 | 36 | Yes | EAA65189.1 | (Bauer et al., 2006) |
| *Schwanniomyces etchellsii* | BglI | 96 | 34 | Yes | ACF93471.1 | (Pandey & Mishra, 1997) |
| *Cellulomonas biazotea* | Cba | 96 | 41 | Not found | AAC38196.1 | (W. K. R. Wong et al., 1998) |
| *Kluyveromyces marxianus* | KmBglI | 96 | 33 | Yes | ACY95404.1 | (Yoshida et al., 2010) |
| *Volvariella volvacaea* | BglII | 97 | 35 | Yes | AAG59831.1 | (X. Li et al., 2005) |
| *Saccharophagus degradans* | Bgl3C | 98 | 32 | Yes | ABD81934.1 | (H. Zhang et al., 2011) |
| *Saccharopolyspora erythraea* | EryBI | 97 | 35 | Yes | CAA74702.1 | (Jakeman & Sadeghi-Khomami, 2011) |
| *Streptomyces antibioticus* | OleR | 92 | 37 | Yes | AAC12650.1 | (Quiros et al., 1998) |
| *Aeromicrobium erythreum* | EryBI | 94 | 37 | Yes | AAU93797.1 | (Reeves et al., 2008) |
| *Streptomyces venezuelae* | DesR | 95 | 35 | Yes | ACR54627.1 | (Zmudka et al., 2013) |
| *Spirochaeta thermophila* | STHERM_  c14600 | 76 | 44 | Not found | ADN02400.1 | (Angelov et al., 2011) |
| *Dictyoglomus turgidum* | Dtur_0219 | 75 | 41 | Not found | ACK41548.1 | (Kim et al., 2011) |
| *Herpetosiphon aurantiacus* | HaGH03 | 76 | 43 | Not found | ABX04075.1 | (R.-F. Wang et al., 2015) |
| *Hungateiclostridium thermocellum* | BglB | 74 | 40 | Not found | ABN52488.1 | (Romaniec et al., 1993) |
| *Paenibacillus xylanilyticus* | BglA | 79 | 38 | Not found | AFC68969.1 | (D.-J. Park et al., 2013) |
| *Thermoclostridium stercorarium* | Bgl3Z | 78 | 39 | Not found | CAB08072.1 | (Adelsberger et al., 2004) |
| *Acetivibrio thermocellus* | Bgl3B | 74 | 39 | Not found | CAA33665.1 | (Gräbnitz et al., 1989) |
| *Xhantomonas citri* | XAC4231 | 89 | 32 | Yes | AAM39066.1 | (Vieira et al., 2021) |
| *Cellulomonas fimi* | Bgl3C | 77 | 44 | Not found | AEE47485.1 | (Gao & Wakarchuk, 2014) |
| *Paenibacillus sp. TS12* | GH3 | 78 | 38 | Not found | BAC16750.1 | (Sumida et al., 2002) |
| Uncultured bacterium | AS-Esc6 | 77 | 41 | Not found | AHG23300.1 | (Biver et al., 2014) |
| *Prevotella bryantii* | Xyl3A | 97 | 26 | Yes | ADD92014.1 | (Dodd et al., 2010) |
| *Prevotella ruminicola* | GH3 | 98 | 27 | Yes | ACN78955.1 | (Dodd et al., 2009) |
| *Bacteroides intestinalis* | GH3 | 95 | 26 | Yes | EDV05842.1 | (Hong et al., 2014) |
| *Bacteroides intestinalis* | BACINT_  01042 | 92 | 27 | Yes | ZP_03013483.1 | (Pereira et al., 2021) |
| *Prevotella bryantii* | Xyl3C | 89 | 27 | Yes | ADD92016.1 | (Dodd et al., 2010) |
