# Supplementary material for: Structure and Function of BcpE2, the Most Promiscuous GH3-Family Glucose Scavenging Beta-Glucosidase
Source: mBio. 2022 Aug 1;13(4):e00935-22. doi: 10.1128/mbio.00935-22 (PMC9426481; doi:10.1128/mbio.00935-22)
Supplement: FIG S2 [file mbio.00935-22-s0002.docx]

**Supplementary Figure S2. Phylogeny of BcpE2 and its closest characterized GH3-family β-glucosidases**

BcpE2 of *S*. *scabiei* is part of the clade that includes Bgl3B of *Cellulomonas fimi*, Cba from *Cellulomonas biazotea*, Gluc3M of *Martelella mediterranea*, and Cbg1 from *Agrobacterium tumefaciens*. Bgl3B of *Cellulomonas fimi*, and Cba from *Cellulomonas biazotea* are the closest partially characterized non-*Streptomyces* actinobacterial GH3s and have been reported to be active on gentiobiose and cellobiose as natural substrates, respectively (Gao & Wakarchuk, 2014; W. K. R. Wong et al., 1998). BcpE2 also shares a common ancestor with Gluc3M, a cold-active and alkali-stable β-Glucosidase from the Gram-negative rhizobiaceae *Martelella mediterranea*, and with the β-Glucosidase Cbg1 from *Agrobacterium tumefaciens*. Cbg1 is the second closest described homologue though it only has 39% and 55% of AA identity and similarity, respectively, compared to BcpE2. Interestingly, Cbg1 is involved in the virulence induction of some *A. tumefaciens* strains targeting Douglas fir trees (*Pseudotsuga menziesii*) by hydrolyzing the monolignol glucoside coniferin (Castle et al., 1992; Morris & Morris, 1990). Moreover, Cbg1, like BcpE2, is not properly active on cellobiose (Castle et al., 1992; Deflandre et al., 2020) but was instead reported to be active on salicin and arbutin. Beyond the synthetic substrates pNPβG and 4-Nitrophenyl β-D-galactopyranoside (pNPβGal), Gluc3M also exhibited significant activities toward salicin, and konjac powder (glucomannan) (Mao et al., 2010). As Cbg1 hydrolyzes the monolignol glucoside coniferin (Castle et al., 1992), the two other monolignol glucosides syringin and p-coumaryl alcohol 4-O-glucoside were also included as candidate substrates. The coumarin heteroside esculin, which is hydrolyzed by the fungal enzyme - KmBglI GH3 enzyme (Yoshida et al., 2010) - belonging to the phylogenetic clade adjacent to the clade of BcpE2 (Figure S2) was selected as well. The coumarin heteroside scopoline was included because it is a new substrate hydrolyzed by BglC of *S*. *scabiei* (Deflandre et al. 2022). BcpE2 of *S*. *scabiei* 87-22 was also suggested by KEGG pathway as candidate beta-glucosidase possibly involved in cyanoamino acid metabolism (https://www.genome.jp/kegg-bin/show_pathway?scb00460+SCAB_64101). Two cyanogenic glucosides were thus also tested as possible targets of BcpE2, *i*.*e*., amygdalin and linamarin. The glycone moiety of amygdalin is the disaccharide gentiobiose and its aglycone part is mandelonitrile, the cyanohydrin of benzaldehyde; linamarin is a glucoside of acetone cyanohydrin. These cyanide-bearing heterosides are plant phytoanticipins whose activation requires the action of a β-glucosidase to release the toxic aglycone moiety from the glycosidic residue. Finally, the natural plant anthocyanidin pigment cyanin, and the synthetic aryl-β-glucoside substrate 4-methylumbelliferyl-β-D-glucoside (4-MUG) were also tested.


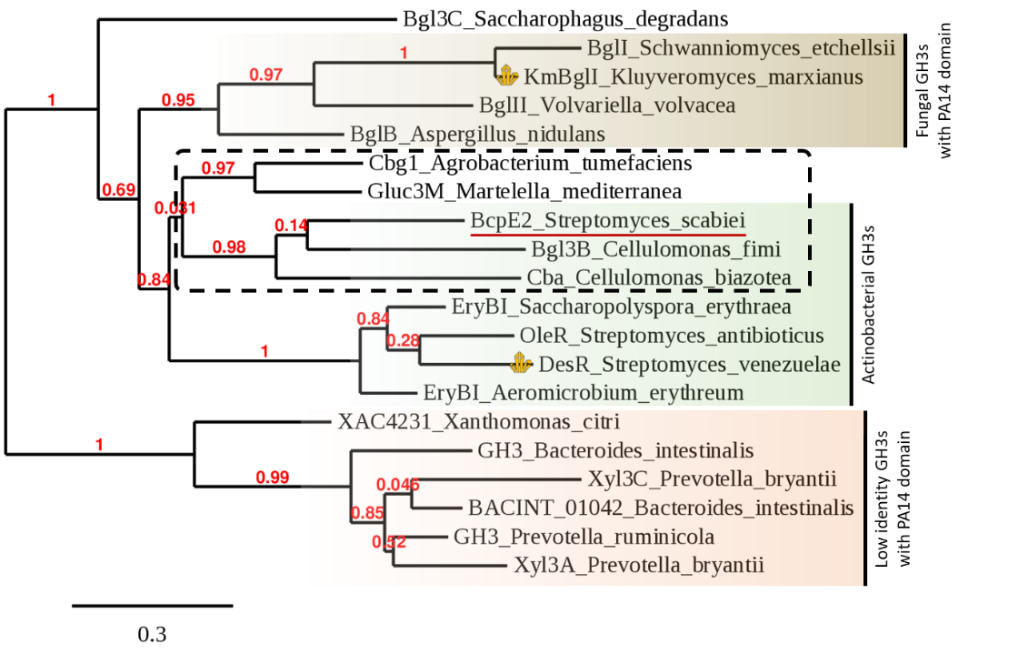


**Figure S2. Phylogeny of BcpE2 and its closest characterized GH3-family β-glucosidases in order to identify possible candidate substrates of BcpE2.** The 19 closest characterized bacterial and fungal GH3-family beta-glucosidase have been selected based on BLASTp score and/or high query coverage. The dotted square delineates the clade with the closest characterized homologues of BcpE2.
