# Supplementary material for: Structure and Function of BcpE2, the Most Promiscuous GH3-Family Glucose Scavenging Beta-Glucosidase
Source: mBio. 2022 Aug 1;13(4):e00935-22. doi: 10.1128/mbio.00935-22 (PMC9426481; doi:10.1128/mbio.00935-22)
Supplement: FIG S3 [file mbio.00935-22-s0003.docx]

**Supplementary Figure S3. Determination of the pH and temperature optima of BcpE2.**

The enzyme was obtained as BcpE2-His_6_ by heterologous production in *E*. *coli* and subsequent purification by Nickel affinity chromatography as previously described (Deflandre et al., 2020). BcpE2-His_6_ shows neutral and mesophilic pH and temperature parameters, with optimal activities displayed around pH 6.5-7.5 and 35-40°C, respectively Figure S3). The optimal pH window was relatively narrow, since the enzyme displayed about 60% of its maximal activity at pH values only 0.5 above or below the 6.5-7.5 range (Figure S3, left panel). This pH range is in line with the cytoplasmic compartmentalization of BcpE2 in *S*. *scabiei*. Below 20°C and above 45°C, the activity rapidly dropped under 50% of the optimal activity (Figure S3, right panel). See the materials and methods section for the detailed protocol.


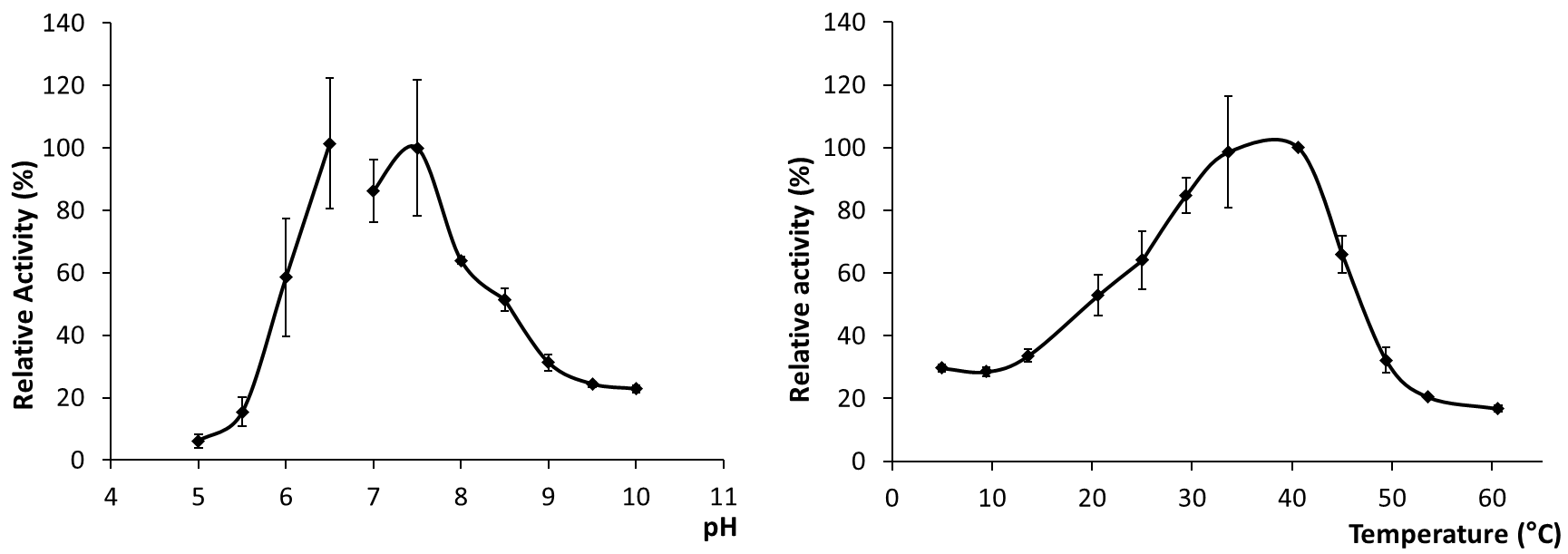


**Figure S3. Determination of the pH and temperature optima of BcpE2.** Relative activity assays with pNPβG as substrate, normalized to the maximal value measured in each assay. The influence of the pH (left panel) was assessed by increments of 0.5 from pH 5 to pH 10, and the temperature (right panel) was assessed by increments of 5°C from 5 to 60°C.
