# Supplementary material for: Structure and Function of BcpE2, the Most Promiscuous GH3-Family Glucose Scavenging Beta-Glucosidase
Source: mBio. 2022 Aug 1;13(4):e00935-22. doi: 10.1128/mbio.00935-22 (PMC9426481; doi:10.1128/mbio.00935-22)
Supplement: TABLE S2 [file mbio.00935-22-s0005.docx]

**Supplementary Table S2**

**Transitions selected for MRM relative quantification of BglC/BcpE2 β-glucosidases**

| **Peptide** | **Precursor ion mass (m/z)** | **CE (V)** | **Fragment y-ion mass (m/z)** |
| --- | --- | --- | --- |
| BglC | | | |
| LVDELLAK | 450.7737^++^ | 16 | \| 787.456^+^ \| \| --- \| \| 688.3876^+^ \| \| 573.3606^+^ \| |
| TDPVASLR | 429.7376^++^ | 15 | \| 642.3933^+^ \| \| --- \| \| 545.3406^+^ \| \| 446.2722^+^ \| |
| BcpE2 | | | |
| AGVLLAQEAR | 514.2984^++^ | 18 | \| 800.4625^+^ \| \| --- \| \| 687.3784^+^ \| \| 574.2944^+^ \| |
| DASGTVIGTR | 488.7565^++^ | 17 | \| 703.4097^+^ \| \| --- \| \| 646.3883^+^ \| \| 545.3406^+^ \| |
| AADTAVVVVATTER | 701.8805^++^ | 25 | \| 973.5677^+^ \| \| --- \| \| 874.4993^+^ \| \| 775.4308^+^ \| |
| BSA | | | |
| AEFVEVTK | 461.7477^++^ | 16 | \| 722.4083^+^ \| \| --- \| \| 575.3399^+^ \| \| 476.2715^+^ \| |
| QTALVELLK | 507.8133^++^ | 18 | \| 785.5131^+^ \| \| --- \| \| 714.476^+^ \| \| 601.3919^+^ \| |
| Phos B | | | |
| VFADYEEYVK | 631.8006^++^ | 22 | \| 945.42^+^ \| \| --- \| \| 830.3931^+^ \| \| 667.3297^+^ \| |
| LLSYVDDEAFIR | 720.8721^++^ | 26 | \| 964.4734^+^ \| \| --- \| \| 865.405^+^ \| \| 750.3781^+^ \| |
| VLYPNDNFFEGK | 721.8512^++^ | 26 | \| 1067.479^+^ \| \| --- \| \| 970.4265^+^ \| \| 856.3836^+^ \| \| 741.3566^+^ \| |

**Detailed protocol of the targeted proteomic approach.**

Fractions collected from the anion exchange chromatography (AXC) were subjected to trichloroacetic acid (TCA) precipitation. The AXC fractions were mixed with 100% (w/v) TCA solution (Sigma-Aldrich) (4:1), the proteins were precipitated overnight at 4°C and collected by centrifugation (16,000g; 30 min, 4°C). The protein pellet was washed twice with ice-cold acetone and solubilized in 50 mM ammonium bicarbonate containing 2 M urea. Protein concentrations were estimated by Bradford’s method (Coomassie Plus Protein Assay kit, Pierce). Dried protein (10 µg) was solubilized in 2 M urea/50mM NH_4_HCO_3_ spiked with bovine serum albumin (MS-grade protein standard) (1:250) (Thermo-Scientific), and denatured by heating to 80°C for 10 min. The solution was subsequently reduced with 5 mM dithiothreitol (Sigma-Aldrich) for 10 min at 60°C and alkylated with 15 mM iodoacetamide for 20 min at RT in the dark before digestion with trypsin (Promega, Madison, USA) overnight at 37°C (1:50 w/w). Acidified digested samples were desalted using OMIX C18 pipette tips (Agilent). The desalted peptides were dried under vacuum and dissolved in 0.1% formic acid, 3% ACN and 10 fmol/µl phosphorylase B (Hi3 Phos B Standard, Waters).

For BglC and BcpE2 identification by MRM, samples (0.5 µg) were injected onto an ultraperformance liquid chromatography (UPLC) M-class system (Waters) and trapped on a 300 µm x 50 mm, 5 µm, 100 Å Acquity UPLC M-Class Symmetry C18 Trap Colum (Waters). The washing step on the trap column was performed for 2 min with 3% B at a flow rate of 15 µL/min, with solvent A 0.1% HCOOH in H_2_O (Biosolve) and solvent B 0.1% HCOOH in ACN (Biosolve). Subsequently, the peptides were separated on a 150 µm x 100 mm, 1.8 µm HSS T3, iKey separation device in 10 min at a flow rate of 2 µL/min using a linear solvent B gradient (3-50%). The separated peptides were introduced into the IonKey source coupled to a Waters Xevo TQ-S triple-quadrupole mass spectrometer for detection of the analytes in the positive-ion mode (ESI+). The MRM mode with transitions of selected proteotypic peptides at a set cone voltage and different collision energies for each precursor, was used for detection, normalization (BSA) and MS performance check (Phos B) (Figure S3). Capillary voltage was set at 3.5kV, the cone voltage at 35V and the source temperature at 120°C. In the collision cell, argon was introduced at a flow rate of 0.15 mL/min. Data were acquired with the developed MRM mode (MassLynx 4.1), subsequently uploaded into Skyline (Pino et al., 2020) for data analysis, and, subjected to a Savitsky-Golay Smoothing, the total area under the curve (AUC) for each peptide was calculated and normalized to BSA.
